# Supplementary figures and images for: Undergraduate GPA does not predict success in PhD programs for cohorts of MS students at two minority-serving institutions
Source: PLoS One. 2026 Feb 2;21(2):e0330005. doi: 10.1371/journal.pone.0330005 (PMC12863499; doi:10.1371/journal.pone.0330005)

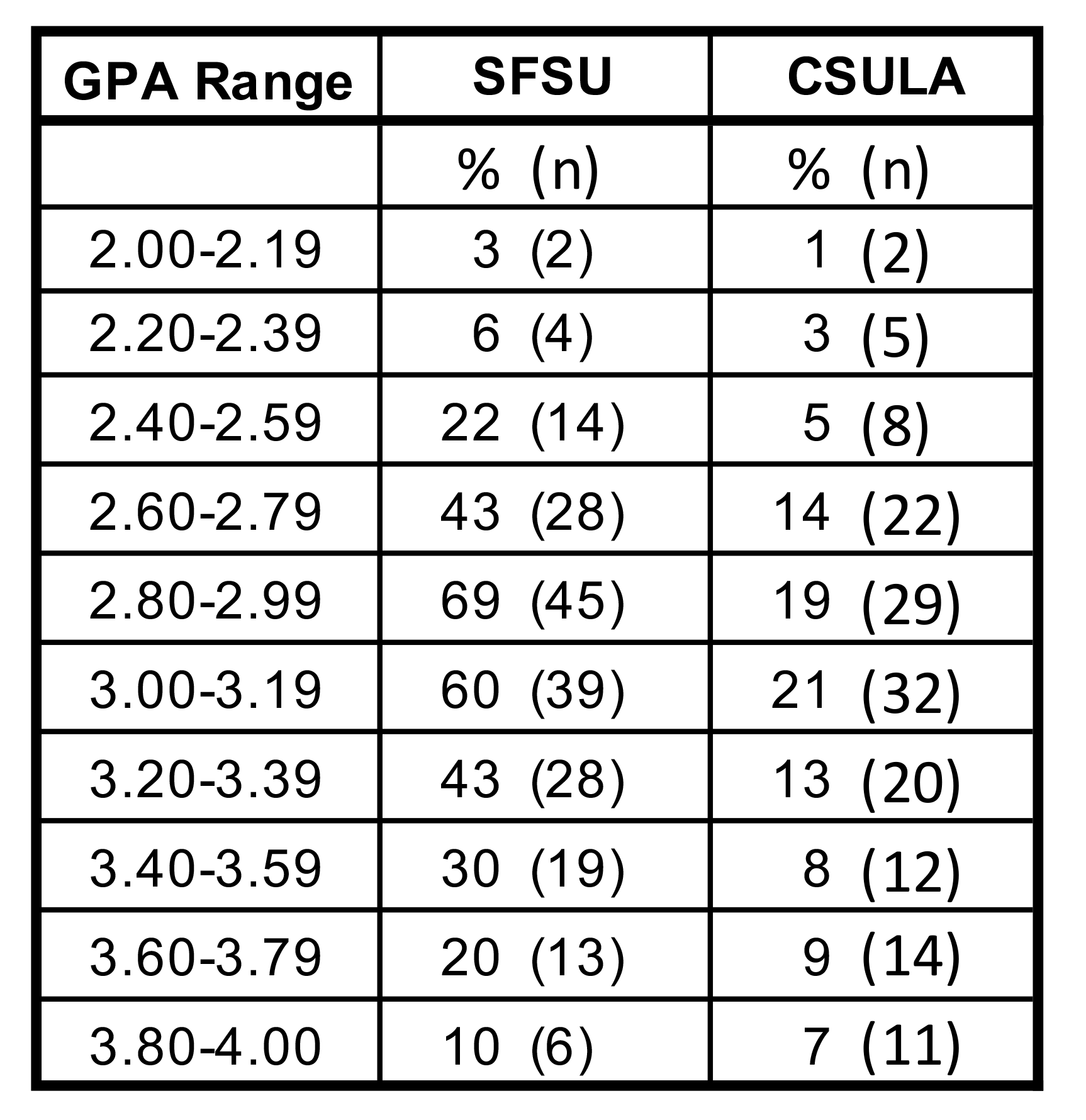

Supplement: S1 Table — (TIF) [file pone.0330005.s001.tif]

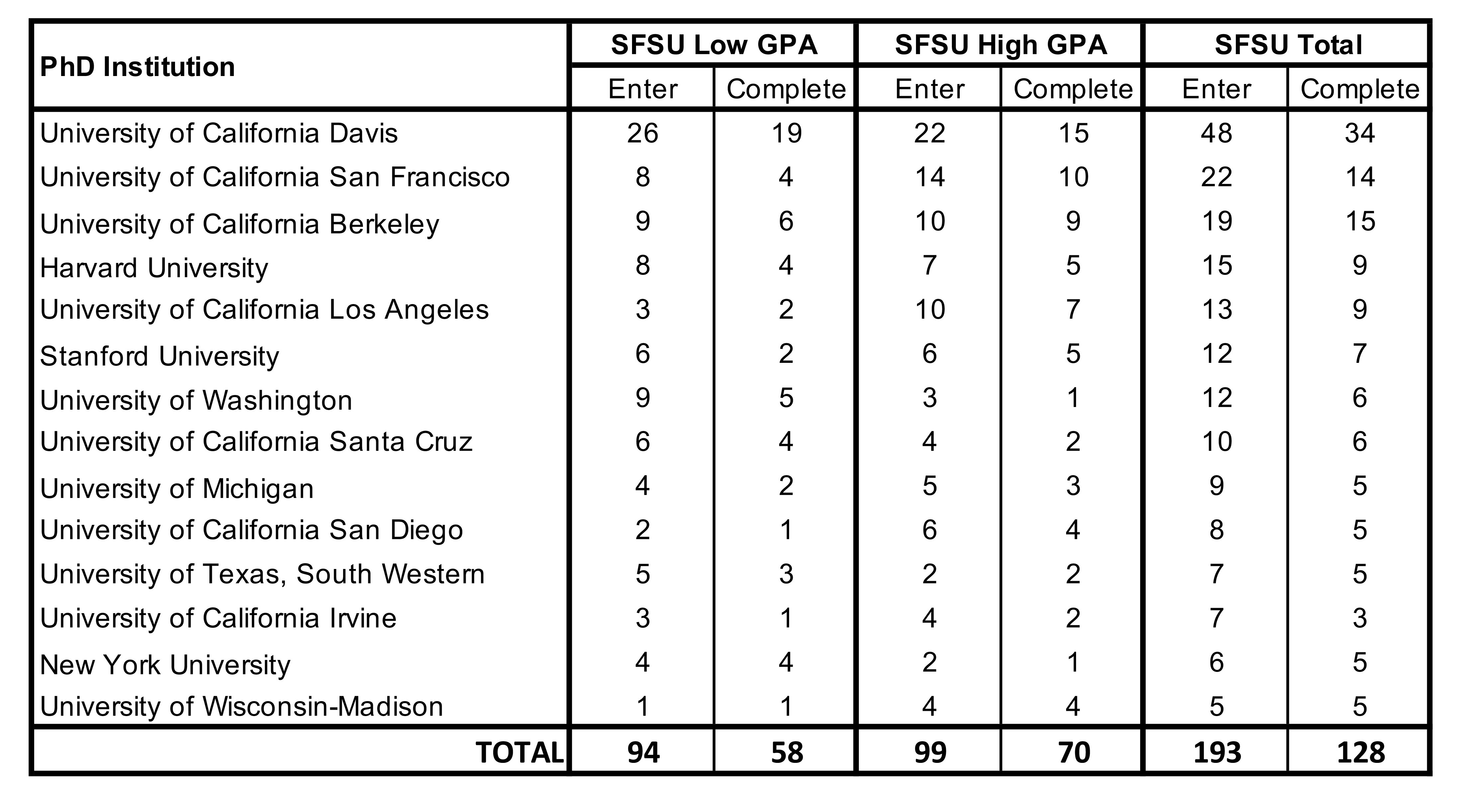

Supplement: S2 Table — Completion rates are lower than entry rates, since some students are still in progress and some (<7%) withdrew. *OTHER institutions (in order of number of acceptances) include: Albert Einstein School of Medicine, Utah State U., U. Arizona, Washington U., Emory U., U. Florida, U. Southern California, Johns Hopkins U., Northwestern U., U. Maryland, U. Montana, U. Tennessee, U.T. San Antonio, Washington State U., Arizona State U., Baylor Medical College, Boston U., Brown U., Texas A&M, U. South Florida, U. Utah, U.C. Merced, U.N.C. Chapel Hill, California Institute of Technology, Cambridge U., College of William & Mary, Columbia U., Cornell U., Dartmouth U., Drexel U., Eastern Virginia Medical School, Kobe U., Loma Linda U., Massachusetts College of Pharmacology, Memorial Sloan Kettering, Pennsylvania State U., Portland State U., Ross U., Seton Hall U., State U. of New York Stony Brook, Temple U., Tulane U., Université Cote d’Azur, U. Alabama, U.C. Riverside, U.C. Santa Barbara, U. Chicago, U. Georgia, U. Hawaii, Manoa, U. Illinois, Urbana-Champaign, U. Iowa, U. Melbourne, U. Missouri, U.T. Health Center Houston, U.T. Austin, U. Pacific, U. Virginia, US Air Force Institute of Technology, Victoria University of Wellington, City University of New York, Florida State U., George Mason U., Georgia Institute of Technology, HHS Center for Disease Control, U. Buffalo, U. Colorado Boulder, U. Nevada Reno, U. North Texas, U. Oregon, Virginia Tech U., Yale U. (TIF) [file pone.0330005.s002.tif]

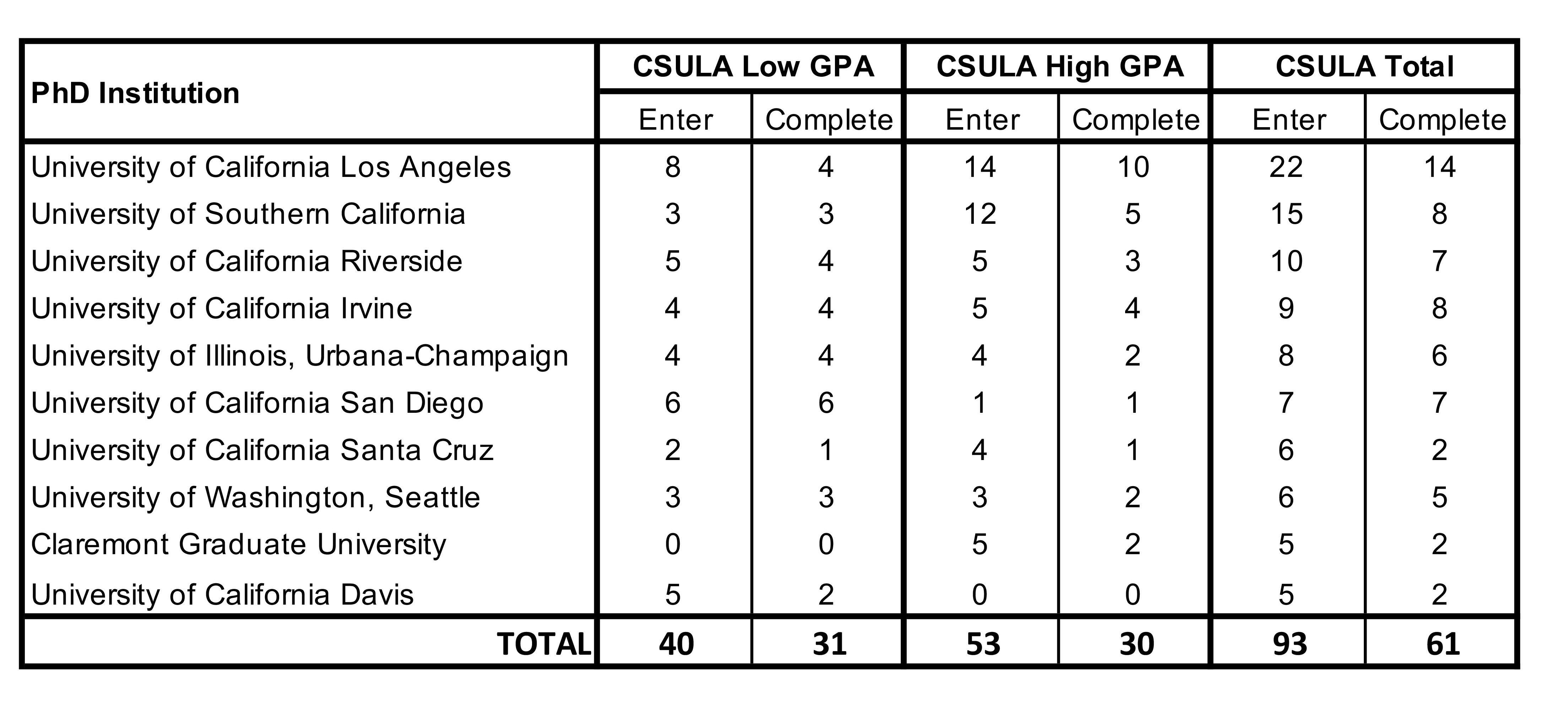

Supplement: S3 Table — Completion rates are lower than entry rates, since some students are still in progress and some (<20%) withdrew. *OTHER institutions (in order of number of acceptances) include: City of Hope National Medical Center, U. of Michigan Ann Arbor, New Mexico State U., Stanford U., U. Arizona, U.C. Berkeley, U.C. Merced, U. Colorado Boulder, U.N.C. Chapel Hill, U. Pittsburgh, Alabama State U. Tuscaloosa, Albert Einstein School of Medicine, Arizona State U., Azusa Pacific U., Baylor U., California Institute of Technology, Columbia U., Cornell U., Georgia Institute of Technology, Georgia State U., Harvard U., Indiana U. School of Medicine, James Cook U. (Australia), Louisiana State U., Massachusetts Institute of Technology, Utah State U., U. Arizona, Washington U., Emory U., U. Florida, U. Southern California, Johns Hopkins U., Max Planck Institute for Astronomy (Germany), Michigan State U., Mississippi State U., New York U., Northeastern U., Sackler Institute for Comparative Genomics, American Museum of Natural History, Scripps Research Institute, La Jolla, Temple U., Texas A&M, U. Alabama Birmingham U. Cincinnati, U. Colorado, Denver, U. Florida, U. Kentucky, U. Massachusetts Amherst, U. of Texas Austin, U. Wisconsin Madison, Vanderbilt U., Western Michigan U., Yale U. (TIF) [file pone.0330005.s003.tif]

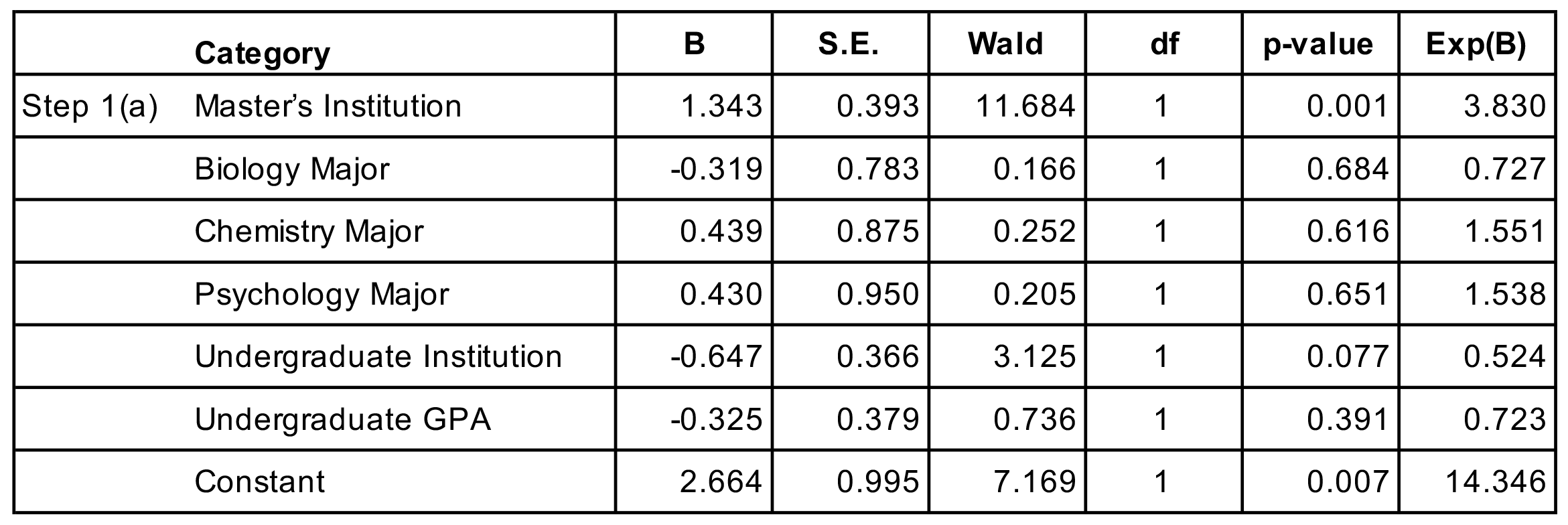

Supplement: S4 Table — (TIF) [file pone.0330005.s004.tif]

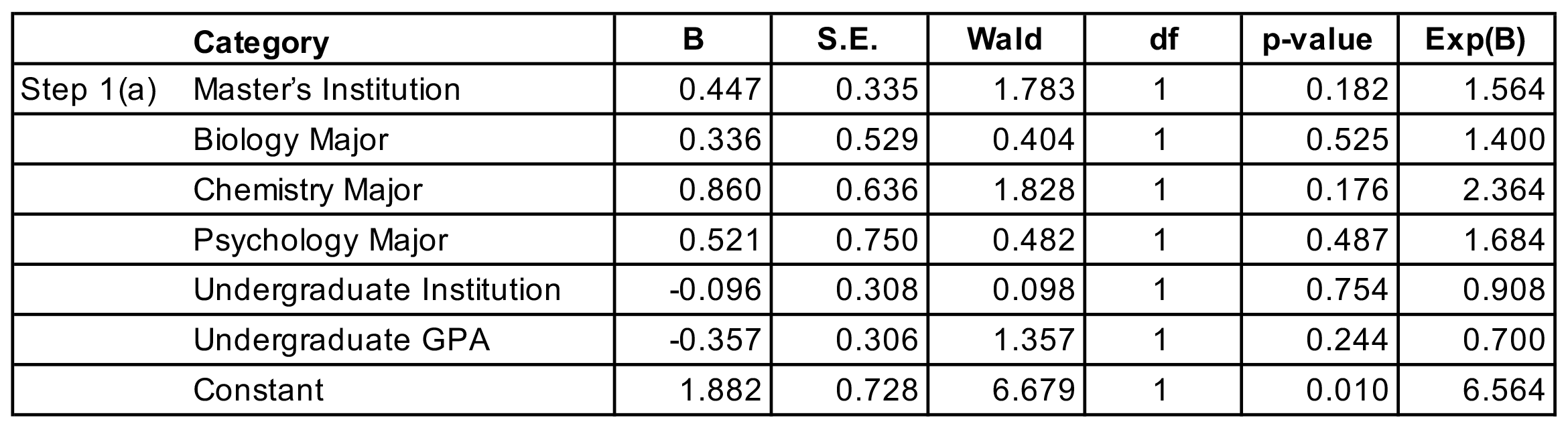

Supplement: S5 Table — (TIF) [file pone.0330005.s005.tif]

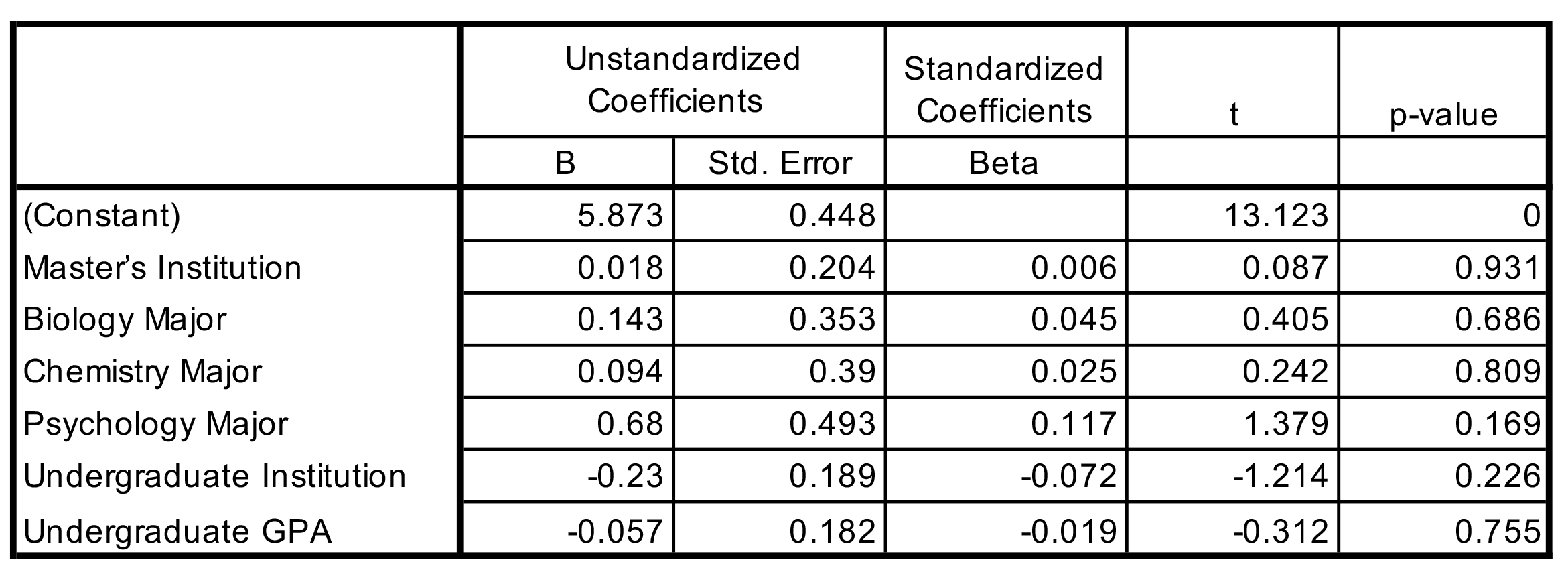

Supplement: S6 Table — (TIF) [file pone.0330005.s006.tif]
